# Community health volunteers could help improve access to and use of essential health services by communities in LMICs: an umbrella review

**DOI:** 10.1093/heapol/czy094

**Published:** 2018-12-24

**Authors:** Mirkuzie Woldie, Garumma Tolu Feyissa, Bitiya Admasu, Kalkidan Hassen, Kirstin Mitchell, Susannah Mayhew, Martin McKee, Dina Balabanova

**Affiliations:** 1 Department of Health Policy and Management, Jimma University, Jimma, Ethiopia; 2 Department of Global Health and Population, Harvard T.H. Chan School of Public Health, Boston, U.S.A; 3 Department of Health, Behaviour and Society, Jimma University, Jimma, Ethiopia; 4 Department of Population and Family Health, Jimma University, Jimma, Ethiopia; 5 University of Glasgow; 6 London School of Hygiene and Tropical Medicine, London, UK

**Keywords:** Low- and middle-income countries, community health volunteers, health services, access, utilization

## Abstract

A number of primary studies and systematic reviews focused on the contribution of community health workers (CHWs) in the delivery of essential health services. In many countries, a cadre of informal health workers also provide services on a volunteer basis [community health volunteers (CHV)], but there has been no synthesis of studies investigating their role and potential contribution across a range of health conditions; most existing studies are narrowly focused on a single condition. As this cadre grows in importance, there is a need to examine the evidence on whether and how CHVs can improve access to and use of essential health services in low- and middle-income countries (LMICs). We report an umbrella review of systematic reviews, searching PubMed, the Cochrane library, the database of abstracts of reviews of effects (DARE), EMBASE, ProQuest dissertation and theses, the Campbell library and DOPHER. We considered a review as ‘systematic’ if it had an explicit search strategy with qualitative or quantitative summaries of data. We used the Joanna Briggs Institute (JBI) critical appraisal assessment checklist to assess methodological quality. A data extraction format prepared a priori was used to extract data. Findings were synthesized narratively. Of 422 records initially found by the search strategy, we identified 39 systematic reviews eligible for inclusion. Most concluded that services provided by CHVs were not inferior to those provided by other health workers, and sometimes better. However, CHVs performed less well in more complex tasks such as diagnosis and counselling. Their performance could be strengthened by regular supportive supervision, in-service training and adequate logistical support, as well as a high level of community ownership. The use of CHVs in the delivery of selected health services for population groups with limited access, particularly in LMICs, appears promising. However, success requires careful implementation, strong policy backing and continual support by their managers.

**Key Messages**

- Community health volunteers (CHVs) are lay individuals of varied background, coming from, or based in the communities they serve, who have received brief training on a health problem they have volunteered to engage with.
- It is evident that CHVs have the potential to supplement the formal health system in the struggle to achieve UHC in low- and middle-income countries (LMICs).
- Preventive, promotive and curative health services provided by CHVs were as good as, or in some cases better than those who are formally employed as health workers.
- In-service training, financial incentives, infrastructural support and supplies, appropriate monitoring, regular supportive supervision and evaluation, and integration of CHV programmes into the formal healthcare system were found to be facilitators of success.
- Lack of regular supervision, limited training, lack of clear definition of roles, too many vertical programmes and insufficient resources were key barriers to success of volunteer-led health programmes.

## Introduction

The burden of disease in low- and middle-income countries (LMICs) is changing rapidly. Progress in tackling infectious and nutritional diseases is threatened by a combination of interlinked factors such as climate change and conflict (GBD 2016 Causes of Death Collaborators, 2017), as well as an increasing burden of non-communicable disease (Harper and Armelagos, 2010; Defo, 2014). This dual burden of infectious and non-communicable diseases (Remais *et al.*, 2013) will require innovative responses and sustained investment in the core building blocks of health systems (GBD 2016 SDG Collaborators, 2017), if the sustainable development goals (SDGs) are to be achieved.

Health workers are critical to addressing these complex challenges. However, low-income countries face a particular challenge in recruiting and retaining health workers (World Health Organization, 2016), now considered the major ‘critical constraint’ to the achievement of health and development goals (Anyangwe and Mtonga, 2007; Kanchanachitra *et al.*, 2011; Beladi *et al.*, 2015; World Health Organization, 2016). Shortages of health workers are impeding progress with essential, life-saving interventions such as childhood immunization, safe childbirth, and access to prevention and treatment for HIV/AIDS, malaria and tuberculosis (Anyangwe and Mtonga, 2007; Conway *et al.*, 2008; Truth, 2013). There is currently an estimated shortage of 7.2 million skilled health workers, projected to exceed 18 million by 2030 (World Health Organization, 2016). Nearly half of this deficit, totalling 3.4 million (47%), is in South-East Asia, with 1.8 million (25%) in Africa. Worldwide, 57 countries have been identified as facing ‘critical shortages’, 36 of which are in Africa. Moreover, the overall numbers conceal marked imbalances within countries, especially in rural areas (Lehmann *et al.*, 2008). The situation has been exacerbated as priority disease programmes compete for more healthcare workers than ever (Chen *et al.*, 2004), at a time when the pull factors of health systems in industrialized countries have grown (Beladi *et al.*, 2015). The need to expand and sustain essential health system functions calls for a broader range of health cadres contributing in a variety of ways.

Traditionally, community members such as traditional birth attendants have filled some of these gaps. However, lay people, with varying degrees of training, are increasingly being brought within the formal health system (Witmer *et al.*, 1995; Lehmann and Sanders, 2007), from small-scale community-based initiatives to national programmes. This development has attracted the attention of researchers, with findings synthesized in a series of systematic reviews looking at, for example, their role, effectiveness, and barriers and facilitators to their work (Witmer *et al.*, 1995). It has been challenging to synthesize learning from these initiatives, not least because these workers go under different names, are recruited in different ways, and have different experiences. For instance, volunteers have been variously defined as frontline workers, lay health workers, health volunteers, community health workers (CHWs), non-specialist healthcare providers and village health agents, among the more common terms (van Ginneken *et al.*, 2013). The scope of their work also varies; ranging from vaccination, bed net distribution, prenatal care and care for chronic diseases like AIDS and tuberculosis (Koon *et al.*, 2013; Saeterdal *et al.*, 2014; Scott *et al.*, 2015; Tripathi *et al.*, 2016).

Several studies have reported that CHWs, including lay health workers, provide several advantages compared with their professional counterparts. They may find it easier to communicate with the community and gain trust from their patients; they can enhance cultural relevance of health materials and information; they may be able to shape the healthcare system to suit their community needs and they can be cost-effective extensions of the health system (Gilmore and McAuliffe, 2013). Thus, community health volunteers (CHVs) are often considered as interposed between communities and the formal health system. They are seen as a means to ‘reach the last mile’ when implementing programmes, removing barriers to healthcare within the community (Lehmann and Sanders, 2007; Berthold, 2016).

Reports from different settings advocate greater use of lay health workers to support understaffed health systems (Raphael *et al.*, 2013; Geldsetzer et al, 2018). However, there is not, to our knowledge, a systematic attempt to understand the roles and contributions of volunteers, as opposed to other lay workers, across the range of health conditions; existing reviews having a narrow focus on specific health conditions. Consequently, our approach recognizes the diverse roles that CHVs, like other CHWs, assume, in different settings (Olaniran *et al.*, 2017), as well as the diversity of settings they work in, the populations they serve (such as urban or rural) and or the conditions they respond to, such as maternal health. For us, the core issue is that they are volunteers, which we define as individuals delivering a health-related service to the community who do not receive a regular salary and/or hold a formal position within the health system. We contend that their status as CHVs may differentiate them, in various ways, from those who are paid, affiliated with and accountable to health system institutions, so that their experiences may offer some lessons that, with care, can be applied in different settings. This study is in response to growing interest in the use of volunteers to assist in provision of essential PHC services across LMIC countries. Our premise is that a synthesis of evidence on their roles and evidence of their effectiveness in improving access to and use of essential health services (often primary healthcare), while charting the barriers and facilitators related to their work, will be advantageous to policy and practice. Consequently, we report the findings of an umbrella review of CHV programmes in LMICs.

We ask three questions: What are the roles played by CHVs? How effective are CHVs in improving access and use of health services compared with other health workers? What are the barriers and facilitators influencing the success of volunteer-led health programmes?

## Methods

This review used a standardized protocol prepared according to the Preferred Reporting Items for Systematic reviews and Meta-Analyses (PRISMA) guidelines (Moher *et al.*, 2009). The protocol was registered with PROSPERO (PROSPERO 2016 CRD42016039361).

### Inclusion criteria

#### Participants/population

The population of interest was defined as CHVs, male and female, who live and work in rural and urban communities of LMICs (based on the World Bank definition), who are involved in any kind of health-related activities, and who are not part of the formal health system. The review was limited to the community volunteers who are not paid regular salaries and who do not possess formal certification required of health professionals. Those workers paid by and affiliated with the health system (e.g. through training and supervision) are referred to as CHWs, a different cadre, and excluded from the review.

The literature often fails to distinguish between CHWs and CHVs, thus complicating the selection of the papers. We have strictly applied the defining criteria that CHVs do not receive a regular salary and/or hold a formal position within the health system. In cases where the reviews reported on both CHWs and CHVs, we extracted only data relating to what the CHVs did, their tasks within health programmes and their impacts. We have included papers reporting on volunteer-led health programmes irrespective of the duration of the health programmes, and type or intensity of engagement of the volunteers.

#### Interventions or exposures of interest

We included papers reporting on involvement of CHVs as the bridge between the formal health system and the community, and their contribution to delivery of a range of preventive, promotive and curative health services. In practice, these are related to maternal and child health, infectious diseases and non-communicable diseases. Hence, the interventions could be related to any health activities as long as these occurred at the community level with the engagement of volunteers.

#### Phenomenon of interest

The phenomena of interest were the various roles undertaken by CHVs and facilitators and barriers affecting their activities.

#### Comparator(s)/control

Our focus was on reviews of studies where the role of CHVs was compared, either over time or cross-sectionally, with situations in which there were no CHVs, including where services are delivered by formally certified health professionals. However, reviews that included relevant descriptive or observational studies were retained to provide context where relevant.

#### Type of reviews included

This review included peer-reviewed systematic reviews of both qualitative and quantitative studies.

*Context:* Studies conducted in LMICs in healthcare institutions, in the community and at homes were considered.

*Outcomes: Primary outcomes:* Utilization of essential health services (such as immunization, family planning, health information, treatment for malaria and TB and others).

*Secondary outcomes:* Programme coverage (family planning coverage, immunization coverage, and others), mortality rate, and morbidity rate.

### Search strategy

The search was conducted by GTF and MW in PubMed, the Cochrane library, the Database of Abstracts of Reviews of Effects (DARE), EMBASE, ProQuest dissertation and theses, the Campbell library and DOPHER. Only studies published in the English language and in peer-reviewed journals were included. The initial search was conducted between July 23 and August 15, 2016 with an updated search carried out on June 8, 2018. The search strategy, described below, was used in PubMed. The initial and updated searches in the other databases used similar terms and limits.

*(*(“Community Health Workers”[Mesh] OR “Volunteers”[Mesh]) OR (“Community health workers”[TIAB] OR “Community health volunteers”[TIAB] OR “Village health workers”[TIAB] OR “Community health aides”[TIAB] OR “Lay health workers”[All Fields])) AND *(*(“Low and middle income countries”[TIAB] OR “developing countries”[TIAB] OR “Sub-Saharan Countries”[TIAB] OR “Asian countries”[TIAB] OR “Latin American Countries”[TIAB] OR Africa[TIAB] OR “LMICs”[TIAB] OR “Low income countries”[TIAB] OR “Middle income countries”[TIAB]) OR (“Developing Countries”[Mesh] OR “Asia”[Mesh] OR “Europe, Eastern”[Mesh] OR “Latin America”[Mesh] OR “Africa”[Mesh])) AND *(*Review[ptyp] AND (“2000/01/01”[PDAT]: “2018/05/31”[PDAT]) AND “humans”[MeSH Terms] AND English[lang])

### Study selection

This umbrella review was limited to systematic reviews, defined as those with explicit inclusion criteria, search strategies, critical appraisal and qualitative or quantitative summaries of data from the primary studies included. Initially, we read titles and abstracts of the reviews and retained those articles describing health programmes involving CHVs in a LMIC health system.

### Critical appraisal for quality assessment

The Joanna Briggs Institute (JBI) critical appraisal assessment checklist for systematic reviews (Aromataris *et al.*, 2015) was used to critically appraise the methodological quality of the retrieved systematic reviews. The assessment checklist was modified to include one additional item (Were there methods to minimize errors in data extraction?) and had 11 questions with a label as Y = yes, N = no, UC = unclear. We included only reviews which scored ‘yes’ for at least 7 of the 11 questions (63.6%). Methodological quality assessment was conducted by two independent reviewers (K.H. and M.W.) with disagreements resolved by discussion or involvement of a third reviewer (G.T.F.) as required.

### Data extraction

A data extraction form was developed by operationalizing the research questions to generate a set of key dimensions. Data extraction was performed by one of the authors (K.H.) and cross checked by two other research team members (M.W. and G.T.F.). The data extracted included specific information on authors, year of publication, participant characteristics, description of the nature of CHVs, the health programme, degree of comprehensiveness of sources searched, number and type of studies included, criteria for recruitment and appraisal of CHVs, relationship of CHVs with the health system, and impact of CHVs on access, utilization and community engagement. We also extracted data on the roles and activities of the volunteers, barriers and facilitators related to implementation, resources used by the CHVs and outcomes assessed. Finally, we noted the key conclusions or findings reported by the included systematic reviews. This deductive approach provided necessary flexibility as the extraction categories were sufficiently broad to allow inclusion of unexpected findings. Decisions on what to include were based on relevance to the research questions.

### Data syntheses

Due to the heterogeneity of the outcomes, populations served and features of the CHV programme, it was not possible to undertake a meta-analysis. Data were therefore synthesized narratively under themes based on our questions.

## Findings

**Figure 1.**
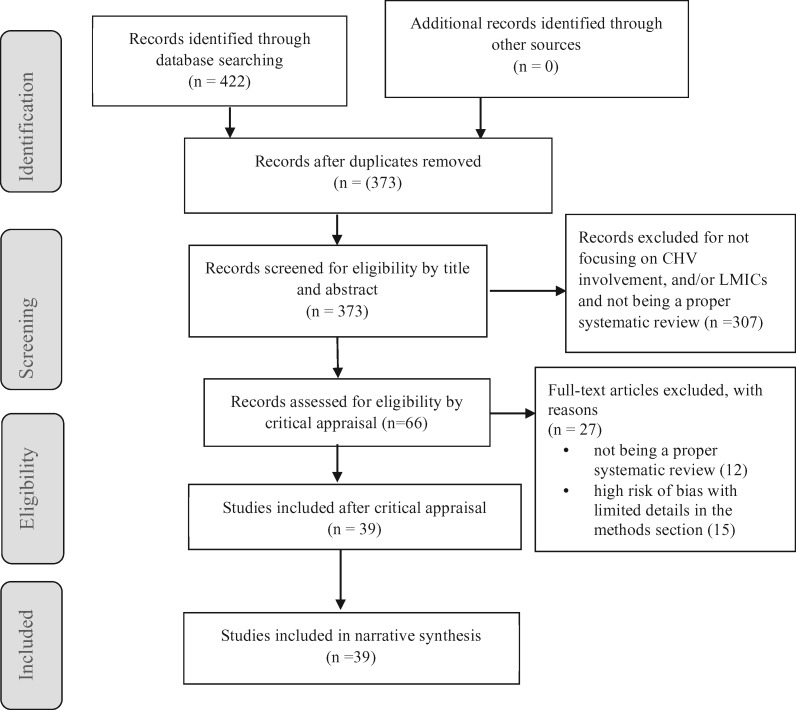
Study selection process (Moher *et al.*, 2009)

The initial search yielded 422 records. After removing duplicates (49 articles), we found 373 records eligible for initial assessment. Following review of titles and abstracts, 72 records met the inclusion criteria and were retained for methodological appraisal. Further reading of the main sections of the papers identified six reviews inappropriate for inclusion (two were duplicates not identified previously, one was not from an LMIC and the other three did not meet the inclusion criteria). This resulted in retention of 66 reviews for methodological quality assessment, after which 27 were excluded. The main reasons were: lack of any objective methodology to satisfy the requirements of a systematic review (12 records) and very high risk of bias with limited details in the Methods section (15 records). Finally, 39 records were included (Figure 1).

### Characteristics of included systematic reviews

More than half of the 39 reviews were published during 2014–2018 while only 7 were published before 2013. Eleven were Cochrane reviews and 28 included randomized controlled trials (RCTs) and cluster randomized controlled trials (cRCTs) alone or combined with other designs. The median number of primary studies included in the reviews was 17 (range 1–106). Search strategies were comprehensive in 38 of the reviews included and the majority (28/39) examined the role of CHVs in maternal and child health (MCH) services and various chronic conditions (Table 1).

**Table 1.** Characteristics of included systematic reviews (*n* = 39)

Year of publication	Number of articles
2007–2012	7
2013	8
2014–2016	13
2017–2017	11
**Country of corresponding author**	
High-income countries	18
LMICs	21
**Publisher of review**	
Non-Cochrane review	28
Cochrane review	11
**Type of study designs included by the review**	
RCT or clustered RCT only	15
RCT and others^a^	13
Observational and analytical designs	3
Mixed methods, qualitative and quantitative	7
Economic evaluations	1
**Sources searched**	
Comprehensive^b^	37
Limited^c^	2
**Number of articles included**	
1–5	4
6–12	14
15–32	12
38–60	6
Others^d^	3
**Target disease/condition**	
MCH services	13
Chronic care^e^	15
Malaria/fever	6
Others^e^	5

a Others include qualitative designs, pre–post evaluations, cohort, post intervention only, interrupted time series, non-randomized control trials.

b All major databases including EMBASE, Medline, Google Scholar and Cochrane Library are included.

c A single database or government or institutional databases are included.

d One review included 94 studies, another one included 106 studies and a third one did not report the number of studies included.

e *Others* include screening of serious illnesses, screening for blindness and serious visual impairment and staffing PHC units and *chronic care* includes DOTs for TB, Buruli ulcer, HIV/AIDS, diabetes, non-communicable diseases, sexual violence and mental illness.

### Description and roles of CHVs

The titles or names given to CHVs (CHWs with no regular salaries) vary across health systems. A review by Shipton *et al.* reported titles such as Accredited Social Health Activists (ASHA), Shasthya Shebika, village midwive, CHWs (Shipton *et al.*, 2017). A review of 32 studies reported that few details were provided in the primary studies on identities of the community or lay health workers (de Vries and Pool, 2017). Similarly, not all of the reviews included contained explicit descriptions of the CHVs involved. When these were provided, the nature of definitions of CHVs varied widely, from brief descriptions to elaborated definitions. The former included, for example, ‘local community members who had no formal education in health care’ (Vouking *et al.*, 2013) or characteristics of those involved, such as mothers, parents and family members, community leaders, drug sellers, students, teachers, members of women’s groups, religious leaders and other lay persons (Ryman *et al.*, 2008; Saeterdal *et al.*, 2014; Boyce and O'Meara, 2017). Among the latter was the reviewed by Kane *et al.*, who defined them as ‘members of the communities where they work … selected by the communities … answerable to the communities for their activities [and] supported by the health system but not necessarily a part of its organization, and have shorter training than professional workers’ (Kane *et al.*, 2010).

Vries and Pool used a broad definition: ‘a heterogeneous group of lay people trained to promote health among their peers in communities’ (de Vries and Pool, 2017). They used the term ‘Key Informants (KIs) to describe CHVs trained to identify children with blindness and severe visual impairment, defining them as ‘community members, who, after very brief training, are expected to network widely to identify children [with blindness or sever visual impairment] in remote rural areas’ (du Toit *et al.*, 2017). In the review by Gatuguta *et al.*, the term volunteer was used interchangeably with ‘community health worker’. The authors reported that volunteers or CHWs included in their review were defined according to the World Health Organization (WHO) which states ‘community health workers are community-based workers who are members of the communities where they work, selected by their communities, have received limited training but are not professional health workers. They are supported by the health system while not necessarily being a part of its organization’ (Gatuguta *et al.*, 2017). Box 1 provides a list of other characteristics of the CHVs.

The roles of CHVs ranged from mere providers of education intended to encourage uptake of care or facilitate behavioural change among community members, to drug distribution and counsellors of patients in their community. The most common role played by the CHVs was awareness raising by informing and educating about communicable diseases and maternal and child health problems (Volmink and Garner, 2007; Okwundu *et al.*, 2013; Smith Paintain *et al.*, 2014; Okebe and Eisenhut, 2014; Feyissa *et al.*, 2015; Gogia and Sachdev, 2016; Nkonki *et al.*, 2017). Several reviews also reported that volunteers engaged in screening, diagnosis and treatment of certain infectious diseases (Hopkins *et al.*, 2007; Bateganya *et al.*, 2010; Okwundu *et al.*, 2013; Okebe and Eisenhut, 2014; Petersen *et al.*, 2014; Smith Paintain *et al.*, 2014; Boyce and O'Meara, 2017). Involvement of the volunteers in mental health and other non-communicable conditions was also reported in a few systematic reviews (van Ginneken *et al.*, 2013; Mutamba *et al.*, 2013; Gatuguta *et al.*, 2017; Jeet *et al.*, 2017; Kaselitz *et al.*, 2017). Details of the wide range of roles assumed are presented in Table 2.

**Table 2.** Diseases/health conditions targeted and roles played by CHVs in LMICs

Disease/health condition targeted	Role of CHWs
Fever/malaria/pneumonia (Hopkins *et al.*, 2007; Okwundu *et al.*, 2013; Okebe and Eisenhut, 2014; Smith Paintain *et al.*, 2014; Boyce and O'Meara, 2017)	Screening of febrile patients [including the conduct of rapid diagnostic test for malaria parasite (RDT) at community and provision of drugs. Treat malaria presumptively or after a positive malaria RDT. Conduct home management of malaria. Rectal drug administration
HIV/AIDS care and support (Bateganya *et al.*, 2010; Mdege *et al.*, 2013; Petersen *et al.*, 2014; Feyissa *et al.*, 2015; Kennedy *et al.*, 2017; Hall *et al.*, 2017)	Lay counsellors offering counselling or behavioural change interventions, e.g. psychological therapies, psycho-education, adherence support and motivational interviewing
	HIV testing service using rapid diagnostic test kits, drug distribution, home visits, outreach activities, health education and counselling. Emotional support, making arrangements for rides to clinics, providing soap and other basic needs, counselling and encouragement to improve retention in HIV care
Tuberculosis (Volmink and Garner, 2007; Bateganya *et al.*, 2010; Hou *et al.*, 2012; Wright *et al.*, 2015; Feyissa *et al.*, 2015)	Health education, regular follow-up, psychological counselling, medication management (DOT)
Buruli ulcer (Vouking *et al.*, 2013)	Curative or preventive care in the control of Buruli ulcer
Mental disorders (Mutamba *et al.*, 2013; van Ginneken *et al.*, 2013; Gatuguta *et al.*, 2017)	Medical and psychological service and interventions delivered in the community. Emotional and social support, psychotherapy and counselling
	Support healthcare service to survivors of sexual violence: Raising awareness, identifying cases, treatment, providing community feedback to healthcare workers at health facilities and providing psychosocial support including individual and group counselling of survivors based in the community. Crisis telephone calls, accompanied survivors to hospitals and the police, provided emotional support and education as well as assisted clinicians in tasks related to managing survivors such as prioritizing treatment, setting up appointments and follow-up at the facilities
Family planning (Scott *et al.*, 2015)	Provided birth control pills and condoms; provided health education
Maternal and child health (Gogia *et al.*, 2011; Gilmore and McAuliffe, 2013; Glenton *et al.*, 2013; Gogia and Sachdev, 2016; Tripathi *et al.*, 2016; Nkonki *et al.*, 2017; Shipton *et al.*, 2017; du Toit *et al.*, 2017)	Promotion of antenatal care; health education and/or counselling regarding desirable practices, during pregnancy; promotion of delivery in a hospital or at home by a skilled birth attendant; education about safe and/or clean delivery practices
	Promotion of optimal neonatal care practices such as exclusive breastfeeding, keeping the baby warm and hygienic cord care; education to improve care-giver recognition of life-threatening neonatal problems and healthcare seeking behaviours; home visit, risk screening and identification of signs of severe neonatal illness
	Identification of children with blindness and severe visual impairment
Emergency obstetric care (Ni Bhuinneain and McCarthy, 2015)	Community interventions that encourage emergency obstetric and neonatal care readiness at family and informal care level. Awareness raising on maternal health problems: anaemia, mal-presentation, retained placenta-obstructed labour and postpartum haemorrhage
Immunization services (Ryman *et al.*, 2008; Saeterdal *et al.*, 2014)	Involved in informing and educating, mobilization and tracking of target populations
Adolescent health services (Koon *et al.*, 2013; Kew *et al.*, 2017)	None specific, any adolescent health service delivered by the healthcare system. Lay-led and peer-support intervention for adolescents with asthma
Non-communicable disease control and prevention (Jeet *et al.*, 2017; Kaselitz *et al.*, 2017)	Health education/health promotion (life style modification advice) for diabetes, cancer, cardiovascular diseases and stroke prevention

### Criteria for the selection of CHVs

Only 13 of the 39 reviews reported on the selection processes for CHVs. The most basic criterion was that volunteers must be living in the community they serve and should be approved by the community (Kane *et al.*, 2010; Koon *et al.*, 2013; Scott *et al.*, 2015). Beyond that, selection criteria were often implicit. Thus, Vouking *et al.*, noted how selection of volunteers is underpinned by the ‘cultural, political and social contexts of the programme area’, with volunteers usually being ‘those that are most acceptable to the community’ (Vouking *et al.*, 2013). Other reviews described quite elementary criteria, such as basic literacy, availability, accessibility, and a willingness to volunteer and serve (Smith Paintain *et al.*, 2014). Beyond that, the criteria were often specific to the roles being undertaken. Thus, CHVs undertaking home visits to identify seriously ill infants required ‘primary education that enables them to read, write and do simple mathematical calculations’ (Tripathi *et al.*, 2016). CHVs in adolescent health programmes had to be between 18 and 40 years of age (Koon *et al.*, 2013). On the other hand, in a review of CHVs in mental health, they were described as ‘any relative or friend of any age who defined themselves [as a] caregiver’ (van Ginneken *et al.*, 2013). Other reviews noted wide variations in the prerequisites, recruitment, training, supervision and workload of community volunteers (Gilmore and McAuliffe, 2013) and selection criteria were inconsistent (Glenton *et al.*, 2013). The latter review summarized attributes expected of volunteers as: being respected and trusted in the community, being married or having children and having particular personal traits such as communication skills, life experience, a willingness to learn and an eagerness to work. In a review focusing on the role of volunteers in providing support health services for survivors of sexual violence, specific criteria such as previous training in the provision of reproductive health services, ability to understand the importance of confidentiality and sensitivity, and being already known for supporting individuals dealing with grief, rejection and sexual violence stigma by the community were reported (Gatuguta *et al.*, 2017).

### Contributions to improving access, utilization and health outcomes

Several reviews reported that involvement of CHVs in primary healthcare activities resulted in improved access and utilization of services by the community (Bateganya *et al.*, 2010; Mdege *et al.*, 2013; Mutamba *et al.*, 2013; Petersen *et al.*, 2014; Okebe and Eisenhut, 2014; Hall *et al.*, 2017). Only 16 of the 39 systematic reviews provided any specific comments on the impact of CHVs on community health. Most focused on process measures, such as increased access to essential drugs/services (Ryman *et al.*, 2008; Mdege *et al.*, 2013; Okwundu *et al.*, 2013), improved primary and secondary prevention of mental illness (Mutamba *et al.*, 2013), increased referrals from community to facility-based providers (Smith Paintain *et al.*, 2014), improved timing and adherence of treatment (Hopkins *et al.*, 2007) and raised awareness about a health condition (Ryman *et al.*, 2008; Gilmore and McAuliffe, 2013; Saeterdal *et al.*, 2014; Ni Bhuinneain and McCarthy, 2015). A few described changes in specific population health outcomes, such as reduction in maternal and child mortality (Okwundu *et al.*, 2013; Ni Bhuinneain and McCarthy, 2015; Gogia and Sachdev, 2016). These reviews are summarized in Table 3.

**Table 3.** Outcomes studied and conclusions reached by each systematic review

Authors (year)	Outcomes addressed	Authors conclusions
Mdege *et al.* (2013)	Mortality, AIDS-defining illness, virological outcomes, CD4 cell count, adherence to ART medicines, hospital admissions, clinic visits, toxicity or adverse events, quality of life indicators, costs and cost effectiveness	Non-inferior patient outcomes can be achieved with task shifting from healthcare professionals to lay health workers (LHWs)
Mutamba *et al.* (2013)	Primary: Changes in incidence or prevalence of mental, neurological and substance use (MNS) disorders	LHWs have the potential to provide psychosocial and psychological interventions as part of primary and secondary prevention of MNS disorders in LMICs, but there is currently insufficient robust evidence of effectiveness of LHW-led preventive strategies in this setting
	Secondary: Knowledge and understanding; health status and wellbeing; rate of provision of services	
Ni Bhuinneain and McCarthy (2015)	Optimal maternal emergency obstetric outcome; early detection of mothers at risk	This review did not identify any research on the potential role of the obstetric first-aider/CHV equipped with life-saving essential drugs for haemorrhage and infection. There are inconsistent results about the effect of peer educators on facility birth rates
Okebe and Eisenhut (2014)	All-cause mortality	In rural areas without access to injectable antimalarial rectal artesunate provided by CHVs before transfer to a referral facility probably reduces mortality in severely ill young children compared with referral without treatment
Okwundu *et al.* (2013)	Primary outcomes: All-cause mortality	Home- or community-based interventions which provide antimalarial drugs free of charge probably improve prompt access to antimalarial, and may impact on childhood mortality when implemented in appropriate settings
	Secondary outcomes: Malaria-specific mortality, hospitalizations, severe malaria, treatment with the recommended antimalarial within 24 h, treatment with any antimalarial, parasitaemia, anaemia and adverse events	
Petersen *et al.* (2014)	Not indicated in the inclusion criteria	Within resource-constrained settings, adjunct behaviour changes and psychological services provided by lay counsellors can be harnessed to promote chronic care at primary healthcare level
Ryman *et al.* (2008)	Immunization coverage	Routine immunization programmes in developing countries may be improved through interventions at the community or facility level
Saeterdal *et al.* (2014)	Knowledge on vaccines or preventable diseases: Knowledge on vaccine service delivery, immunization status of child, any other measures of vaccination status in children (e.g. number of vaccine doses received) and unintended adverse effects due to the intervention	Interventions aimed at communities to inform and educate about early childhood vaccination by volunteers may improve attitudes toward vaccination and probably increase vaccination uptake under some circumstances
Scott *et al.* (2015)	Use of contraceptives and changes in knowledge and attitude	Strong evidence exists to promote volunteer-led family planning programmes to improve access to family planning services
Smith Paintain *et al.* (2014)	Drug dose, cure/rate for malaria and cure rate for pneumonia	CHVs are able to provide good quality malaria care including performing procedures such as rapid diagnostic tests. CHVs are able to treat uncomplicated pneumonia although there is a room for improvement, particularly in accurate diagnosis
Tripathi *et al.* (2016)	Successful identification of seriously ill young infants and improved care seeking from health facilities	There was moderate quality evidence that home visits by trained CHVs are associated with improved care seeking for ill young infants to health facilities in resource-limited settings
van Ginneken *et al.* (2013)	Improvement of symptoms (e.g. level of anxiety, depression and psychosis), psychosocial functioning and impairment (e.g. levels of self-esteem, perception of coping, level of dependency, self-care ability) and quality of life outcomes	There is low quality evidence that LHW-led psychological interventions may increase the number of adults who recover from depression or anxiety, or both 2–6 months after intervention
Volmink and Garner (2007)	TB cure rate, treatment completion and development of clinical TB	Trials comparing home observation (community observer or family observer) to clinic or healthcare worker-led observation did not show any difference in TB cure or treatment completion
Willcox *et al.* (2015)	Role of CHVs in staffing health institutions	Staffing is inversely related to level of need, and health worker density is not increasing despite most countries recognize village health workers, traditional healers and traditional birth attendants
Wright *et al.* (2015)	TB treatment success and loss to follow-up	Community-based DOT has a higher treatment success compared with clinic-based DOT
Vouking *et al.* (2013)	Number of cases of Buruli ulcer identified, number of cases referred and confirmed	The involvement of CHVs has a considerable impact on the control of Buruli ulcer by improving community knowledge about the disease, early case detection and referral
Hou *et al.* (2012)	TB cure and treatment completion rates and DOT adherence	Treatment effects of the different types of care providers and quality improvement interventions did not differ significantly
Kane *et al.* (2010)	Effectiveness of CHWs training for improving delivery of child health interventions	Training interventions in the form of knowledge and skills-based completion, health system interventions in the form of setting clear roles and specific responsibilities for the CHVs and ensuring good referral support and mentoring and better positioning of the CHVs (e.g. involvement of the community in the selection, the CHV being a member of the same and being considered as a model) to improve performance of volunteer-led child health interventions
Hopkins *et al.* (2007)	Indicators of malaria morbidity (incidence, severity, parasite rates) and/or mortality	Presumptive treatment of febrile children with pre-packaged anti-malarials in home management of malaria programmes is likely to increase delivery of effective drugs, and improve the timing, adherence, and dosing of treatment
Kok *et al.* (2015)	CHW performance characteristics: self-esteem, motivation, attitudes, competencies, guideline adherence, job satisfaction and capacity to facilitate community agency. End-user level: utilization of services, health-seeking behaviour, adoption of practices promoting health and community empowerment	Contextual factors related to community (most prominently), economy, environment, and health system policy and practice can influence CHW performance and the programmes
Gilmore and McAuliffe (2013)	No restriction on outcomes; generally focused on effectiveness in providing preventive interventions for maternal and child health	CHWs are effective at increasing acceptability of mother-performed practices, such as skin-to-skin care and exclusive breastfeeding
		CHWs are capable of providing interventions beyond their traditional scope and with more intense training, such as those of a psychosocial nature or delivering scheduled intermittent preventive treatment for malaria
		CHWs are effective in delivering health promotion or education, especially with simple, targeted messages
Koon *et al.* (2013)	Adolescent health services	Though few comprehensive evaluations of large-scale CHW programmes exist, there is mixed evidence to support the use of either generalist or specialist CHW models for delivering adolescent health services
Gogia *et al.* (2011)	Neonatal mortality rate (NMR)	Community new-born care through home visitation with/without community mobilization and community participatory action and learning interventions decreased NMR
Glenton *et al.* (2013)	Barriers and facilitators of lay workers in MCH activities	Rather than being seen as a lesser trained health worker, LHWs may represent a different and sometimes preferred type of health worker
		The close relationship between LHWs and recipients is the strength of programmes involving CHVs
Feyissa *et al.* (2015)	Stigma and sexual behaviour	Home-based HIV counselling testing delivered by lay counsellors reduced stigma and risky sexual behaviour
Bateganya *et al.* (2010)	HIV test uptake	Home-based HIV counselling and testing increased the uptake of HIV counselling and testing
Karumbi and Garner (2015)	TB treatment cure and completion rates	Comparison of DOT at home by family members, or CHWs, with DOT by health workers at a health facility showed little or no difference in cure or treatment completion
Gogia and Sachdev *et al.* (2016)	Neonatal and infant death, perinatal mortality, cause-specific mortality including deaths due to neonatal sepsis, tetanus, asphyxia and prematurity	Home-based neonatal care is associated with reductions in neonatal and perinatal mortality (high-quality evidence) in South Asian settings with high neonatal mortality rates and poor access to health facility-based care. Adopting a policy of home-based neonatal care provided by CHWs is justified in such settings
Boyce and O’Meara (2017)	RDT test safety, accuracy and interpretation; appropriate treatment with anti-malarial drugs	RDTs are used safely and effectively by CHW which included teachers and other lay persons
de Vries and Pool (2017)	Community or lay health worker programme effectiveness and sustainability	Most studies provide anecdotal evidence that the community relationship matters to programme outcomes and attention to traditional roles and networks improves programme effectiveness
du Toit *et al.* (2017)	Productivity in identifying children with blindness and severe visual impairment	The use of community volunteers and formal health sector workers as key informants in campaigns is more productive and less expensive way of identifying children with blindness and severe visual impairment than survey method
Gatuguta *et al.* (2017)	Provision of support healthcare services to survivors of sexual violence	There is potential for CHVs providing support healthcare services for sexual violence but there is lack of quality evidence on appropriate models, acceptability of the services to survivors and feasibility of delivering the services
Hall *et al.* (2017)	Barriers and facilitators in interventions for retention in HIV care	Barriers to lay health worker retention intervention effectiveness included high patient caseloads and lack of preparedness in dealing with acute stressors (e.g. patient adverse events and patients moving) and coordination with lay health workers as case managers facilitated effectiveness in retention care
Jeet *et al.* (2017)	Role of CHWs in the prevention and control of non-communicable diseases	Compared with standard care, using CHWs (volunteers included) in health programmes have the potential to be effective in LMICs, particularly for tobacco cessation, blood pressure and diabetes control
Kennedy *et al.* (2017)	Provision of HIV testing services using RTDs	The existing evidence supports allowing lay providers to conduct HIV testing services using RDTs
Kew *et al.* (2017)	Safety and efficacy of lay-led and peer-support interventions for adolescents with asthma	Weak evidence suggests that lay-led and peer-support interventions could lead to a small improvement in asthma-related quality of life for adolescents, benefits for asthma control, exacerbations and medication adherence remain unproven
Nkonki *et al.* (2107)	Economic evaluation of CHW (volunteers include) interventions aimed at improving child health outcomes	There is evidence of cost effectiveness of CHWs interventions in reducing malaria, asthma and mortality of neonates and children under 5 years of age. Other economic evaluation studies show evidence of cost effectiveness in improving exclusive breastfeeding, malnutrition, physical health and psychomotor development in children, and maternal health

The contributions by the volunteers to these improvements include: informing and educating to raise awareness and service uptake, detection and treatment of infectious diseases, scaling-up services while incurring less cost to the health system, and provision of psychosocial support and mental health care. Each of these roles is discussed in the following four sections.

#### Informing and educating

Hall *et al.* reviewed 11 studies and concluded that ‘lay health workers provided excellent health education and counselling and outreach activities, and their involvement was acceptable to most patients’ (Hall *et al.*, 2017). A review of 21 RCTs by Gogia *et al.* reported that CHVs could contribute to better maternal and neonatal health through education of mothers (Gogia *et al.*, 2011). Topics of this health education included antenatal care, safe and clean delivery practices, the importance of skilled birth attendance, exclusive breastfeeding, keeping the baby warm, hygienic cord care and recognition of life-threatening neonatal problems. Ryman *et al.* examined 60 studies, reporting that CHVs could improve immunization services, encourage uptake and take services closer to the communities (Ryman *et al.*, 2008). The main roles implicated in positive change included education, mobilization and tracking of target populations.

Petersen *et al.* concluded that adequately trained, supervised and monitored CHVs could contribute to better management of chronic diseases (Petersen *et al.*, 2014), bridging formal health services and the community, based on community outreach teams. These volunteers screened and identified patients with chronic conditions and followed up non-adherent patients. They also increased community involvement in the health programme. Interventions included behavioural change interventions such as motivational interviewing. However, the authors concluded that fidelity to intended counselling models was sub-optimal.

Jeet *et al.* reported on the role of CHWs (volunteers included) in the prevention and control of non-communicable diseases (Jeet *et al.*, 2017). After reviewing 16 trials they concluded, ‘Compared with standard care, using CHWs [volunteers included] in health programmes have the potential to be effective in LMICs, particularly for tobacco cessation, blood pressure and diabetes control’. More specifically, another review reported that a structured health education by peer supporters (volunteers) improved A1c and systolic blood pressure levels among diabetic patients better than those in professionally led groups (Kaselitz *et al.*, 2017).

A review of CHVs providing health education to mothers (Gogia *et al.*, 2011) found 21 RCTs and concluded that they could influence attitudes and practices positively. Another review, with 19 studies, concluded that CHV programmes increased acceptability of maternal practices such as skin-to-skin care and exclusive breastfeeding (Gilmore and McAuliffe, 2013). Other reviews of CHVs found that community interventions to provide education on childhood immunization could improve attitudes toward them and, in some circumstances, improve uptake (Ryman *et al.*, 2008; Saeterdal *et al.*, 2014).

Ni *et al.* reported on 22 RCTs of programmes using CHVs to improve obstetric and neonatal care readiness (Ni Bhuinneain and McCarthy, 2015), finding that they could reduce obstetric complications. They did so by raising awareness about maternal health problems, such as anaemia, mal-presentation, retained placenta, obstructed labour and postpartum haemorrhage. Another review concluded that CHVs are ‘effective in delivering health promotion or education, especially with simple, targeted messages’ (Gilmore and McAuliffe, 2013). However, others noted a lack of evidence on whether CHVs engaged in awareness raising interventions influenced rates of institutional delivery or whether they could provide emergency life-saving obstetric interventions (Ni Bhuinneain and McCarthy, 2015).

Tripathi reviewed the use of trained CHVs to identify seriously ill young infants during home visits found seven RCTs (Tripathi *et al.*, 2016), concluding that there was moderate evidence that they increased health-seeking behaviour. One review of community-based new-born care included five RCTs and two cohort studies, finding an association with reduced neonatal mortality rates (Ni Bhuinneain and McCarthy, 2015). Similarly, another review which included five trials recommended adoption of a policy of home-based neonatal care provided by CHWs based on high-quality evidence for reduction of neonatal and perinatal mortality (Gogia and Sachdev, 2016).

#### Detection and treatment of infectious diseases

Okebe *et al.* found a single RCT in which CHVs improved access to treatment of malaria by providing rectal artesunate to adults and children with severe malaria in rural areas without access to injectable antimalarial drugs before transfer to a referral facility (Okebe and Eisenhut, 2014). Vouking *et al.* examined the role of CHVs in management of Buruli ulcer, caused by mycobacterium ulcerans (Vouking *et al.*, 2013), identifying 17 observational studies. CHVs were found to be effective in detecting and treating ulcer with appropriate supervision and infrastructure support from the formal health system.

A review of CHVs in management of malaria in children concluded that they had potential to reduce mortality due to malaria, but this was based on a single RCT (Okebe and Eisenhut, 2014). Another, which included 10 trials (Okwundu *et al.*, 2013), reached the same conclusion, with trained, monitored and supervised CHVs improving prompt access to anti-malarial drugs.

Smith *et al.* reviewed 43 studies (RCTs and pre–post studies) on the role of CHVs in management of fever, with distribution of drugs at community level and improved referral from the community to facility-based providers (Smith Paintain *et al.*, 2014) and concluded that while their ability to make accurate diagnoses was imperfect, they could treat most cases of malaria and pneumonia. Likewise, a review of 18 RCTs concluded that CHVs treating febrile children with pre-packaged anti-malarials in home management of malaria (HMM) programmes could improve delivery of effective drugs, and enhance timing, adherence and dosing of treatment (Hopkins *et al.*, 2007).

A review of six RCTs found that CHVs acting as distributors of ARTs achieved patient outcomes that were as good as those with salaried health workers (Mdege *et al.*, 2013). Similarly, two reviews on the effectiveness of CHVs (family or CHWs) in the provision of directly observed short course therapy (DOT) for tuberculosis showed that outcomes (cure/completion rates) with volunteer-led home-based DOT programmes were not significantly different from facility-based healthcare provider-led programmes (Volmink and Garner, 2007; Karumbi and Garner, 2015). Moreover, another review, of eight (RCT and observational) studies (Wright *et al.*, 2015), concluded that a community-based DOT programme led by community volunteers was more successful than the clinic-based one. A similar finding was reported in a review of 12 (RCTs and pre–post evaluations) studies (Hou *et al.*, 2012).

Gilmore *et al.*, assessing the effectiveness of CHVs in providing preventive interventions in maternal and child health (Gilmore and McAuliffe, 2013) concluded that: ‘[CHVs] are capable of providing interventions beyond their traditional scope and with more intense training, such as those of a psychosocial nature or delivering scheduled intermittent preventive treatment for malaria.’

#### Scaling-up services with less cost to the health system

A review of six RCTs by Mdege *et al.* reported that lay health workers with adequate training, support and supervision, and a monetary/material allowance could increase uptake of anti-retroviral therapy (ART) in home visits (Mdege *et al.*, 2013). This review noted that task shifting from health professionals to CHVs ‘can potentially reduce cost of ART provision without compromising health outcomes’ in the patients.

Others found that CHVs could contribute to more efficient use of health resources (Mdege *et al.*, 2013; Nkonki *et al.*, 2017; du Toit *et al.*, 2017). A review of economic evaluations of CHWs (volunteers included) interventions aimed at improving child health outcomes reported that the interventions were cost effective in reducing malaria, asthma and mortality of neonates and children under 5 years of age; and improving exclusive breastfeeding, malnutrition, physical health and psychomotor development in children, and maternal health (Nkonki *et al.*, 2017).

Scott *et al.* identified 56 studies (RCTs and observational) of CHVs as providers of family planning services (Scott *et al.*, 2015). The authors concluded that CHVs engaged in outreach could increase knowledge and utilization of family planning. CHVs selected by the community could also contribute to scaling up and increased coverage by youth friendly health services (Koon *et al.*, 2013).

A review by du Toit *et al.* found two studies in which CHVs were used as key informants to identify children with blindness and severe visual impairment were more productive (8 and 10 times more, respectively) than workers of the formal health sector (du Toit *et al.*, 2017). In the review by Kennedy *et al.* one RCT, an observational study and three other comparison studies suggested that lay providers achieved similar test quality as trained healthcare providers and increased uptake of HIV tests (Kennedy *et al.*, 2017).

#### Provision of psychosocial support and mental health services

Mutamba *et al.* reviewing 15 studies, including qualitative and quantitative designs, concluded that CHVs could provide effective emotional and social support, psychotherapy and counselling to healthy people and those with mild mental illnesses (Mutamba *et al.*, 2013). Gatuguta *et al.* concluded that there is a potential for CHVs to provide support to healthcare services for survivors of sexual violence and suggested further studies to determine delivery model, feasibility and acceptability of the approach (Gatuguta *et al.*, 2017).

Van Ginneken reviewed 38 studies of CHVs as primary caregivers, offering basic psychotherapy to people with mental illness (van Ginneken *et al.*, 2013), and concluded that CHVs ‘have some promising benefits in improving people’s outcomes for general and perinatal depression, post-traumatic stress disorder and alcohol-use disorders.’ Another review concluded that community-based mental health services provided by volunteers could improve primary and secondary prevention of mental illness (Mutamba *et al.*, 2013). Nevertheless, it noted insufficient robust evidence of effectiveness of volunteer-led preventive strategies in community settings.

### Barriers and facilitators for volunteer-led health interventions

Only 22 of the 39 reviews included identified barriers or facilitators to the work of CHVs. Based on a review of 94 studies, Kok *et al.* developed a taxonomy of five groups of factors that influence the performance of CHV programmes (Kok *et al.*, 2015). These were: (a) *community context* such as socio-cultural factors (socio-cultural norms, values, practices and beliefs, gender roles and norms, and disease-related stigma); safety and security and education and knowledge level of the target group; (b) *economic context*, whereby economic hardship may discourage community members from volunteering; (c) *environmental context*, such as long travel distances, difficult topography and harsh climate; (d) *health system policy*, meaning the impact of human resources policies on incentives and career structures of CHVs, legislative constraints on their scope of work, and the political commitment to them; (e) *health system practice* factors such as how well the health service functions, human resources capacity, level of decision making, costs of health services and governance of the primary healthcare system. We have slightly modified this taxonomy as *community-related factors* (community and environmental context), *volunteer-related factors* (economic context and other CHV characteristics and *health system-related factors* (health system policy and practice). We use this modified framework to structure our findings in the remainder of this section, with the reviews summarized in Table 4.

**Table 4.** Barriers and facilitators of CHV involvement and success in PHC services

	Barriers	Facilitators
Community factors	Limited community ownership (Gilmore and McAuliffe, 2013) Socio-cultural norms, values, practices and beliefs hindering healthcare seeking from CHVs (Kok *et al.*, 2015; Gatuguta *et al.*, 2017) Gender roles and norms that compromise access to and uptake of service by CHVs (Gilmore and McAuliffe, 2013) Disease-related stigma preventing information sharing to CHVs and health-seeking behaviour (Kok *et al.*, 2015) Difficult geography and dispersed settlement with increased travel distance (Kok *et al.*, 2015; Gatuguta *et al.*, 2017) Lack of social recognition and acceptance (Glenton *et al.*, 2013; Gatuguta *et al.*, 2017) Economic hardship (Kok *et al.*, 2015) Negative opinions of healthcare quality or availability (Shipton *et al.*, 2017) Disapproval or lack of support from family members (Shipton *et al.*, 2017)	Involvement in selection and support of CHVs (Kane *et al.*, 2010; Koon *et al.*, 2013; Scott *et al.*, 2015; Tripathi *et al.*, 2016; du Toit *et al.*, 2017) Respect to the volunteers (Ryman *et al.*, 2008) Gender roles and norms favouring interaction between different sexes of client and CHV (Kok *et al.*, 2015) Community participation and ownership (Gilmore and McAuliffe, 2013; de Vries and Pool, 2017) Trust (Gatuguta *et al.*, 2017)
Health system factors	Low or no payment/incentives to volunteers (Scott *et al.*, 2015; Shipton *et al.*, 2017) Lack of supervision (Gogia *et al.*, 2011; Petersen *et al.*, 2014; Smith Paintain *et al.*, 2014; Kok *et al.*, 2015) Limited/insufficient or inconvenient training (Gogia *et al.*, 2011; Gilmore and McAuliffe, 2013; van Ginneken *et al.*, 2013; Shipton *et al.*, 2017) Lack of clear definition of roles (Petersen *et al.*, 2014; Kok *et al.*, 2015) Insufficient resources (Gilmore and McAuliffe, 2013; Kok *et al.*, 2015; Shipton *et al.*, 2017) Lack of clear career pathways (Petersen *et al.*, 2014; Kok *et al.*, 2015) Limited referral pathways (Petersen *et al.*, 2014; Kok *et al.*, 2015) Lack of programme acceptability (Glenton *et al.*, 2013) Lack of programme appropriateness (Glenton *et al.*, 2013) Lack of programme credibility (Glenton *et al.*, 2013) Too many vertical programmes (Kok *et al.*, 2015)	Recognition (Gogia *et al.*, 2011; Petersen *et al.*, 2014; Smith Paintain *et al.*, 2014; Kok *et al.*, 2015) Provision of in-service training (Kane *et al.*, 2010; Petersen *et al.*, 2014) Supportive supervision and mentoring (Kane *et al.*, 2010; Gogia *et al.*, 2011; Mdege *et al.*, 2013; Babu and Babu, 2014; Petersen *et al.*, 2014; Smith Paintain *et al.*, 2014; du Toit *et al.*, 2017) Adequate response for logistical requirements (Petersen *et al.*, 2014) Integration into the formal health system (Kok *et al.*, 2015; Scott *et al.*, 2015) Well-functioning health services (Kane *et al.*, 2010; Kok *et al.*, 2015) Mobile phone use to keep in contact (du Toit *et al.*, 2017)
Volunteer-related factors	Uncertainty on patient outcomes and quality of care (Mdege *et al.*, 2013) Inadequate space and time (too many responsibilities) (Petersen *et al.*, 2014; Gatuguta *et al.*, 2017; Hall *et al.*, 2017) Poor follow-up of patients (Petersen *et al.*, 2014) Economic hardship (Kok *et al.*, 2015) High turnover (Scott *et al.*, 2015) Lack of safety and confidence (Gatuguta *et al.*, 2017) Access to community members (Ryman *et al.*, 2008) Lack of knowledge of the community (Ryman *et al.*, 2008) Income based on drug selling (Kok *et al.*, 2015) Lack of preparedness in dealing with acute stressors (e.g. patient adverse events and patients moving) (Hall *et al.*, 2017) Unmet expectations of recognition from the community (Shipton *et al.*, 2017)	Individual sense of altruism and social recognition (Glenton *et al.*, 2013; Shipton *et al.*, 2017) Individual desire for job satisfaction, nature of responsibilities, incentives and peer support (Shipton *et al.*, 2017) Knowledge gain and career development (Glenton *et al.*, 2013) Feeling of safety and security (Kok *et al.*, 2015) Door-to-door visits (du Toit *et al.*, 2017) Familiarity and shared experiences with population served (Kaselitz *et al.*, 2017)

#### Community-related factors

Community-related barriers to the successful operation of health programmes involving CHVs reported in the systematic reviews ranged from socio-cultural issues such as unfavourable norms, values, practices and beliefs hindering health seeking from CHVs to difficult geography, long travel distance and dispersed settlement of community members (Kok *et al.*, 2015; Olaniran *et al.*, 2017). Shipton *et al.* found that negative opinions about healthcare quality or availability by community members and disapproval or lack of support from family members are key demotivators to volunteers (Shipton *et al.*, 2017). On the other hand, involvement of community members in selection of CHVs, respect and acceptability of the volunteers, and community ownership were seen as facilitating success (Ryman *et al.*, 2008; Kane *et al.*, 2010; Gilmore and McAuliffe, 2013; Koon *et al.*, 2013; Kok *et al.*, 2015; Scott *et al.*, 2015; Tripathi *et al.*, 2016).

However, a review of 32 studies reported that there was ‘minimal inclusion of even basic community level indicators’ to sufficiently understand the influence of community health resources on the effectiveness and sustainability of health programmes led by community or lay health workers. Hence, the authors concluded that ‘most studies provide anecdotal evidence that the community relationship matters to programme outcomes and attention to traditional roles and networks improves programme effectiveness’ (de Vries and Pool, 2017). This review also noted that many of the studies included did not identify the community as the driving force to the volunteer-led health programmes and did not provide any information on the issue of community participation in programme planning.

#### Volunteer-related factors

Volunteer-related facilitators of success include social recognition, feeling of safety and security (Glenton *et al.*, 2013; Kok *et al.*, 2015). More specifically, Glenton *et al.* found that factors including altruism, social recognition, knowledge gain and career development were facilitators of the success of community-based neonatal and maternal health programmes led by CHVs (Glenton *et al.*, 2013). Previous experience on a health project in the community was also reported as facilitator of success (du Toit *et al.*, 2017; de Vries and Pool, 2017). However, de Vries and Pool reported that studies were limited to identifying importance of recruitment in collaboration with communities and mentioning previous experience in health projects is desired but failed to report on its effect on effectiveness of the volunteer-led programmes (de Vries and Pool, 2017).

Other barriers related to the characteristics of CHVs included lack of required knowledge and skill, inadequate space and time, high turnover and dependence on drug selling for income (Ryman *et al.*, 2008; Mdege *et al.*, 2013; Petersen *et al.*, 2014; Scott *et al.*, 2015; Kok *et al.*, 2015). Mdege *et al.* identified uncertainty about what comprised high quality care, both in theory (e.g. official guidelines) and in practice (Mdege *et al.*, 2013). Similarly, a review of volunteer-led chronic care services identified lack of clear definition of the role of lay counsellors, the lack of clear career pathways for advancement for lay counsellors, inadequate counselling space and time, limited referral pathways and poor follow-up of patients counselled as key barriers to success (Petersen *et al.*, 2014).

In their review focusing on CHVs involved in the provision of preventive interventions for maternal and child health, Gilmore and McAuliffe reported that limited community ownership and insufficient resources for volunteers were impediments to success (Gilmore and McAuliffe, 2013). Hence, it is not surprising that the volunteer’s knowledge of the community they work with, the respect they are given by the community and the fact that they have access to community members were identified as facilitators of success in another review (Ryman *et al.*, 2008).

#### Health system-related factors

Lack of supervision, limited training, lack of clear definition of roles, too many vertical programmes and insufficient resources were key barriers to success within the health system, as reported in the reviews (van Ginneken *et al.*, 2013; Gilmore and McAuliffe, 2013; Smith Paintain *et al.*, 2014; Petersen *et al.*, 2014; Kok *et al.*, 2015; Shipton *et al.*, 2017). Insufficient and/or inconvenient training was a particular problem in several of the reviews (Gogia *et al.*, 2011; Gilmore and McAuliffe, 2013; van Ginneken *et al.*, 2013; Shipton *et al.*, 2017). Scott *et al.* indicated that the main challenge of programmes using community volunteers as family planning service providers was the low retention rate of the volunteers because of lower or no payments provided (Scott *et al.*, 2015). Hence, the integration of the service into the formal health system was the main facilitator for success in these programmes. However, Ryman *et al.* identified lack of sustainability as a major challenge in volunteer-led immunization programmes (Ryman *et al.*, 2008). The review by Glenton *et al.* also found that the lack of programme acceptability, appropriateness and credibility were barriers to success.

Health system-related facilitators of success in volunteer-led programmes included recognition, supportive supervision and mentoring, provision of in-service training and adequate response for logistical needs of the volunteers (Kane *et al.*, 2010; Gogia *et al.*, 2011; Mdege *et al.*, 2013; Babu and Babu, 2014; Smith Paintain *et al.*, 2014; Kok *et al.*, 2015). Several reviews have reported that supportive supervision by those in the formal health system was a critical facilitator for the success of the CHV-led programmes while lack of such support from the health system resulted in the failure of the programmes (Gogia *et al.*, 2011; Petersen *et al.*, 2014; Smith Paintain *et al.*, 2014). Petersen *et al.* identified the provision of in-service training and better response for logistical requirements of counselling as major facilitators of these programmes (Petersen *et al.*, 2014). Another review by Mdege *et al.* also highlighted recognition and supportive supervision as major motivational mechanisms (Mdege *et al.*, 2013).

## Discussion

This umbrella review identified, appraised and synthesized systematic reviews on CHVs. It provides an overall picture of the activities of community volunteers in LMICs and the health outcomes to which they contribute. This adds to and extends earlier systematic reviews of the contribution of CHVs in relation to specific health conditions or diseases, or in particular geographic localities or populations. To our knowledge, this is the first umbrella review to assimilate what is known about CHVs in LMIC who have volunteer status. It demonstrates that there is a lack of agreement on definitions of CHVs and their roles and contributions. While Kok *et al.* have argued that CHWs sit between the community and the health system (Kok *et al.*, 2017), our review suggests that CHVs may serve a similar intermediary role while being even closer to their communities.

The reviews describe women and men who willingly engage in the provision of preventive and curative health services to the communities they belong to. They receive no regular payment for their contributions (although they may be compensated for their expenses) and have a relatively brief training/orientation on the health activities they engage in. In addition, they are usually outside the formal health system although they may receive support from it to discharge their functions. Their backgrounds are diverse. Some have no formal education, while others are teachers, students, community leaders and members of civil society organizations. Hence, we suggest that CHVs could be defined as: lay individuals of varied background, coming from, or based in the communities they serve, who have received brief training on a health problem they have volunteered to engage with. They contrast with CHWs by their lack of formal status and are situation outside the health system. However, while not core to this definition, it should also be noted that CHVs often act as multipurpose development workers involved in a variety of community-based work beyond health.

It follows from this definition that CHVs offer a potentially valuable resource for health systems in LMICs that face severe challenges in recruiting and retaining health workers (World Health Organization, 2016) as recruitment of conventional CHWs, who are also close to the communities they serve, are limited by financial constraints. CHVs face few such constraints and can be deployed in greater numbers with minimal cost to the formal health system. This has the potential to improve access to essential health services at community level, but only if the use of CHVs can be shown to be effective. We have shown that they may be able to achieve results that are as good as, or in some cases better than those who are formally employed as health workers. However, this does not mean that CHVs always perform as well as or better than those in the formal health system, so it is important to ensure that any roles envisaged for them are appropriate, which may vary according to context, and that measures known to promote success are adopted.

Importantly, by being outside the formal health system, but with close ties with the community, they may achieve greater trust and corresponding ability to influence those in their communities, not least because they are known to have experienced the same challenges as those they are serving. On the other hand, this position increases the probability that they will experience shortage of resources, lack of supervision and support, and lack of clear career pathways.

CHVs face challenges that conventional CHWs do not, or at least to the same degree. They may face disapproval from family members as their voluntary activity brings no income to the family, thereby adversely influencing retention. CHVs are also prone to role confusion given that they often participate in multiple programmes, lacking clear role definitions.

These considerations raise several questions. The first is how CHVs are recruited. This is an area where the existing reviews have provided only limited information. Some were chosen by their respective communities, using varying, but poorly specified mechanisms, and where criteria are reported, they seem to be quite broad, including being from the community served, willingness to volunteer and knowledge of the community. Most CHVs had only limited literacy and lacked formal certificates of education or training. This would seem to be an area that would benefit from additional research, including assessment of how recruitment, and initial training and induction, relate to performance and retention.

Second, as a priority, where CHVs are used, it should be in settings and roles where they have been found to be effective. These include a broad range of activities related to preventing diseases, treating them, and promoting health, but all at a quite basic level. They can be effective working in a variety of ways, ranging from promoting community mobilization and awareness raising through to specific tasks such as psychotherapy and counselling (Bateganya *et al.*, 2010; Mdege *et al.*, 2013; Mutamba *et al.*, 2013; Ni Bhuinneain and McCarthy, 2015; Feyissa *et al.*, 2015), and provision of immunization, distribution of drugs for malaria, TB and HIV (Bateganya *et al.*, 2010; Feyissa *et al.*, 2015). They can be found in a variety of settings, including health facilities but, especially, homes and the community. Evidence so far points to potential in areas traditionally prioritized by governments, such as maternal and child health and major infectious diseases, such as malaria and TB but, looking ahead, they may be able to contribute more broadly, subject to evaluation of their performance.

Third, skills and training matter. While they perform basic tasks well, they may struggle with more complex activities. Thus, they may fail to achieve fidelity to psychological interventions (Petersen *et al.*, 2014) and lack skills to achieve the same level of diagnostic accuracy as trained health workers (Smith Paintain *et al.*, 2014). While they may be able to raise awareness of issues among expectant mothers, it is not clear whether this translates into greater use of institutional delivery services and nor is there evidence of effectiveness in delivering life-saving obstetric interventions (Ni Bhuinneain and McCarthy, 2015). Thus, it cannot be assumed that the ability to deliver basic interventions shows promise for more complex ones.

Fourth, some things can be done to increase success of CHVs. These include in-service training, financial incentives, infrastructural support and supplies, appropriate monitoring, regular supportive supervision and evaluation, and integration of CHV programmes into the formal healthcare system. It is important to offer clear guidance about their roles and realistic expectations about their prospects for career development. There are also contextual factors that increase the chances of success, including the CHV’s knowledge of their communities, the respect they receive from their communities and their accessibility to community members. Alignment of the attitudes and practices of CHVs with the prevailing culture and expectations of the community seem to improve both access to and uptake of services, especially where the services in question are culturally embedded, such as family planning.

In summary, the roles and performance of CHVs in health programmes vary considerably. However, at least in the roles examined in these reviews, the basic services they provide are often as good as those provided by other health workers. In resource scarce settings, they can make an important contribution to improving access and utilization of primary healthcare services. However, it is essential to recognize their limitations. They are not trained health workers and so cannot be expected to diagnose and treat anything other than the simplest of conditions. This also means that they require training and supervision by those in the formal health system. On the other hand, their proximity to the community coupled with their ability to act as a bridge to existing primary health systems does offer potential, especially where resources are scarce. They must also be adequately equipped and supported. Although some reviews did not report on the resources used by CHVs, most indicated that resources from the formal health system were used by them to discharge their responsibilities.

With renewed concern for universal health coverage (UHC) in the era of the SDGs, the role of CHVs in LMICs seems under-explored. Our findings suggest that CHVs can make a greater contribution to extending coverage, especially to disadvantaged population groups in LMICs where there is a critical shortage of health professionals. However, this demands careful thought by policymakers and health managers. CHVs are not a cheap substitute for adequately trained health workers and, if they are to be successful, even though they are volunteers they must be adequately resourced, with supportive supervision and mentoring, in-service training and adequate logistical support.

There are still many unanswered questions. The existing literature says little about the contribution of CHVs to population health indicators, and especially whether, and in what circumstances, they can achieve results comparable with trained staff. Moreover, the literature on barriers and facilitators for successful roll out of volunteer-led health programmes lacks strong evidence on the nature and characteristics of factors identified. For instance, there are no studies reporting on models of community engagement, frequency and modality of supportive supervision and nature of in-service trainings that optimize success. It is also important to note that although it is claimed that procedures for recruitment and selection of community volunteer matter, something that is intuitive, few studies report on what approaches work in what circumstances.

Furthermore, we have not found much evidence in this overview of empowerment and grassroots initiatives by the CHVs themselves. This is surprising as CHVs are very close to their communities and share the same problems and experiences as their fellow community members. This should allow them to help communities engage in the design, implementation and monitoring of health programmes in their locality.

## Limitations

Our study has some limitations. We included reviews published only in English. However, the comprehensive nature of our search, including all the major databases, means that our search covers all regions in the world. The depth of information available to address some of the review questions was not always optimal in the reviews we have included. However, we are confident that the information we have reported provides an accurate picture of volunteer-led health programmes in LMICs.

Further exploration of the findings, such as meta-analysis, was not possible, because of the diversity of outcomes, study designs and health issues addressed in the reviews we included. Though the systematic reviews have reported both positive and nil effects of CHVs, few attempted to assess publication bias. Moreover, few assessed the strength of evidence for each outcome. Future systematic reviews should assess the strength and quality of evidence using the Grading of Recommendations, Assessment, Development and Evaluation (GRADE) (Schünemann *et al.*, 2013).

## Conclusions

CHVs are lay individuals of varied background, coming from, or based in the communities they serve, who have received brief training on a health problem they have volunteered to engage with. We find sufficient evidence to support use of CHVs in the delivery of certain preventive, promotive and curative services to the community they belong. These include the diagnosis and treatment of malaria, counselling and testing for HIV, distribution of drugs, dissemination of health messages and psychosocial support of some mentally ill people. However, CHVs have not been found to be effective in managing more complex activities. We have also reported on several barriers and facilitators of success related to the community, to health systems and to the volunteers. It is evident that CHVs have the potential to supplement the formal health system in advancing progress to UHC in LMICs. However, there is a need for more studies that examine CHVs and their capabilities to support decision making by policymakers.

**Box 1. Definitions of CHVs**

- ‘_**…**_ are those who receive training, recognized by the health services and national certification authority to perform clearly delineated tasks’ (Mutamba *et al.*, 2013).
- ‘_**…**_ are those who do not have any formal professional or paraprofessional qualifications and are trained to provide health related services’ (Petersen *et al.*, 2014).
- ‘_**…**_ are health care service providers who have typically been trained for a short period of time and lack formal medical training. They often live in the communities they serve and ideally are linked to the formal health system’ (Defo, 2014; Scott *et al.*, 2015).
- ‘_**…**_ are men and women chosen by the community and trained to deal with the health problems of individuals and the community, and to work in close relationship with the health services’ (Conway *et al.*, 2008; Tripathi *et al.*, 2016).
- ‘Lay individuals trained in the particular role of delivering curative or preventive care’ (Anyangwe and Mtonga, 2007; Truth, 2013; Vouking *et al.*, 2013).
- ‘_**…**_ a member of the community who has received some training to promote health care or who carries out some health care services, but is not a professional’ (Nkonki *et al.*, 2017).
- ‘_**…**_ are lay health supporters who are able to establish rapport with patients in the communities they serve in part because of their shared characteristics and experiences_**…**_’ (Kaselitz *et al.*, 2017).
